# How Does the Absence of Job Embeddedness Contribute to Nurses’ Turnover Intention? A Fuzzy‐Set Qualitative Comparative Analysis

**DOI:** 10.1155/jonm/2341935

**Published:** 2026-06-19

**Authors:** Xin Wang, Ming Liu, Jun-E. Zhang, Renli Deng, Mengqi Li, Xiaoyan Jin, Hongxia Dai, Yan Wang, Xin Wang, Angela Yee Man Leung

**Affiliations:** ^1^ Faculty of Health Sciences and Sports, Macao Polytechnic University, Macao, China, mpu.edu.mo; ^2^ Peking University Health Science Center-Macao Polytechnic University Nursing Academy, Macao Polytechnic University, Macao, China, mpu.edu.mo; ^3^ School of Nursing, Sun Yat-Sen University, Guangzhou, China, sysu.edu.cn; ^4^ Department of Nursing, Zunyi Medical University, Zunyi, China, zmc.edu.cn; ^5^ School of Nursing, The Hong Kong Polytechnic University, Hong Kong, China, polyu.edu.hk; ^6^ School of Nursing, Peking University, Beijing, China, pku.edu.cn; ^7^ WHO Collaborating Centre for Community Health Services, The Hong Kong Polytechnic University, Hong Kong, China, polyu.edu.hk; ^8^ Research Institute of Smart Ageing (RISA), The Hong Kong Polytechnic University, Hong Kong, China, polyu.edu.hk

**Keywords:** job embeddedness, nurses, qualitative comparative analysis, turnover intention

## Abstract

**Background:**

Nurse turnover is a significant contributor to the global nursing shortage, and nurses’ turnover intentions are typically caused by a combination of factors. Nurses’ job embeddedness is an important factor influencing nurses’ turnover intentions. However, existing studies have examined only the net effects of the individual constructs on turnover intention, overlooking the combined effects of the job embeddedness dimensions.

**Aim:**

To explore how the dimensions of job embeddedness combine to form configurations that lead to nurses’ turnover intention or no turnover intention.

**Design:**

A cross‐sectional study using fuzzy‐set qualitative comparative analysis.

**Setting(s):**

Six medical institutions in the Hong Kong Special Administrative Region, China.

**Participants:**

From March to June 2023, a total of 232 employed nurses were included using a convenience sampling method.

**Methods:**

The fuzzy‐set qualitative comparative analysis was used to analyze the set relationships between the six dimensions of job embeddedness and turnover intention. Robustness checks and predictive validity analyses, based on set theory methods, were employed to verify the robustness of the condition configurations and their ability to predict the outcome variables. Finally, ordered logit regression was used to examine the associations between the configurations and turnover intention.

**Results:**

Fuzzy‐set qualitative comparative analysis indicated that multiple job embeddedness–related conditions combine to lead to nurses’ turnover intention. Necessity analysis shows that job embeddedness–related conditions are not necessary conditions for the presence or absence of nurses’ turnover intention. Sufficiency analysis identified five configurations contributing to nurses’ turnover intention (solution coverage = 0.525) and six configurations contributing to no turnover intention (solution coverage = 0.668), and these configurations were categorized into six groups. Significant associations were found between these configurations and turnover intention.

**Conclusion:**

The results suggest that the absence of organization fit and organization sacrifice is the core condition that contributes to nurses’ turnover intention. Mutual collaboration and competition between the dimensions of job embeddedness combined to create multiple configurations that prevented nurses’ turnover intention. This study provides new insights and ideas for managers to implement targeted interventions to reduce nurses’ turnover intention in response to these configurations.

## 1. Introduction

Nurses and midwives play a crucial role in global primary healthcare systems, making significant contributions to health improvement and economic development. Increasing the number of nurses will enhance citizens’ life expectancy, which in turn will promote economic growth [1]. However, a global shortage of 4.8 million nurses and midwives is projected by 2030 [2]. This worldwide shortage should be treated as a global health emergency [3], as it profoundly impacts nurse–patient ratios, job stress, burnout, staff retention, patient safety, and the quality of nursing care [4].

Nurse turnover is a major factor contributing to the global nursing shortage, with the International Council of Nurses [3] reporting that the global nurse turnover intention rate has risen to 20% or more, while the annual hospital turnover rate exceeds 10%. The reasons behind nurses’ turnover intentions are multifaceted. Their decision to leave is seldom triggered by a single event or reason and usually results from a combination of challenging situations they face over time [5]. Therefore, a single factor has limited ability to explain actual turnover behavior [6], and interventions or management strategies based on such single factors are often difficult to implement effectively in complex clinical settings, making it challenging to address nurse turnover issues. Turnover intention is often the result of multiple, interacting factors [7]. However, traditional linear regression primarily focuses on the singular, linear relationships between various factors and turnover intention, which makes it insufficient to fully explain the underlying mechanisms that lead to turnover intention. Therefore, qualitative comparative analysis, based on set theory, offers a method for identifying the configurations of conditions that lead to specific outcomes. This approach can reveal the complex interactions among multiple factors and provide new insights for nursing managers in understanding why nurses develop turnover intention.

Job embeddedness can be viewed as a series of reasons that discourage employees from leaving their jobs [6]. It represents a wide range of influences on employee retention and can be described as a “web” that sticks employees to their jobs [6]. This theory categorizes the factors that deter nurses from turnover into six constructs. Understanding how these constructs interact to influence nurses’ turnover intentions can provide deeper insights into why nurses leave their jobs and organizations, thereby enabling the adoption of targeted management strategies to strengthen the nursing staff reserve. Therefore, this study aimed to employ qualitative comparative analysis to explore how multiple factors, through complex interactions, lead to nurses’ turnover intentions based on job embeddedness theory. The findings provide new insights into the underlying mechanisms of turnover intention and inform the development of more targeted, effective retention strategies for administrators.

## 2. Background

Turnover is generally understood as consisting of two distinct concepts: turnover intention and turnover rate [8]. Turnover intention refers to an employee’s tendency or intention to leave their current job or organization at some point [9], while turnover rate is the percentage of employees who leave their current company, organization, or industry relative to the total number of employees [10]. Turnover intention is highly correlated with active exit behavior and is often considered the most direct predictor of turnover rate [10, 11]. Nurse turnover is particularly damaging to the healthcare industry, as the loss of trained, professional nurses not only leads to a permanent decline in productivity but also increases organizational human resource management costs. This, in turn, increases the workload and stress of the remaining active nurses, posing an ongoing, cumulative threat to the sustainability of the nursing workforce [4, 12, 13]. Meanwhile, nurse turnover has exacerbated the imbalance in the nurse‐to‐patient ratio, reducing patient satisfaction and leading to negative patient outcomes [14–16]. Furthermore, nurse turnover is one of the costliest challenges facing healthcare organizations. The economic loss or cost associated with the turnover of a registered nurse is approximately 1.2–1.3 times the average annual salary of a registered nurse in the hospital [17]. Given the adverse effects of nurse shortages and turnover, it is urgent for administrators to identify the reasons for nurses’ turnover and provide interventions to address the issue of nurse’s turnover.

The reasons that contribute to nurses’ turnover intention are well understood [7]. Among these reasons, nurses’ job embeddedness is a key factor in deterring turnover intention, which has been confirmed in a previous meta‐analysis [18]. Highly embedded employees have many tightly connected links, and these different levels of entanglement and embeddedness keep them in their jobs [6]. Engaging employees in their jobs depends on three work‐related forces: link, fit, and sacrifice, which play important roles both on‐the‐job (organization) and off‐the‐job (community). This 3 × 2 matrix presents six dimensions: organization link, organization fit, organization sacrifice, community link, community fit, and community sacrifice. For the detailed conceptual definition of the core concept of job embeddedness, please refer to Table 1.

**TABLE 1:** Definitions of three work‐related forces in job embeddedness.

Concept	Definition
Link	The formal or informal connections between a person and institutions or other people [19].
Fit	An employee’s perceived compatibility or comfort with an organization and its environment [20].
Sacrifice	The perceived cost in material or psychological benefits that an employee loses as a result of leaving the organization [19].

Job embeddedness theory suggests that job embeddedness is not a unified construct. It is comprised of on‐ and off‐the‐job forces that reflect a range of reasons why nurses stay in their jobs. Therefore, rather than viewing job embeddedness as a single factor influencing turnover intention, it is more accurate to consider it as six distinct forces that prevent nurses from leaving their jobs. The majority of existing studies treat job embeddedness as a single concept [19]. This limits policymakers to develop effective management strategies based on this theory, as a single construct cannot adequately capture the specific reasons why nurses may want to quit. Additionally, the complementary and competitive effects between different dimensions of job embeddedness were overlooked. These dimensions may work together to help nurses remain in their jobs [6]. However, nurses often face work–family conflicts, so there may also be competing relationships between community and organization embeddedness [21]. Some existing studies have explored the correlation between multiple dimensions and turnover intention and obtained results that contradict the theory, showing positive correlations between the link dimensions and turnover intention, which remain unexplained [20, 22].

In addition, there is a need to recognize that turnover intention is usually the result of the combined effects of multiple factors, with each nurse leaving for different reasons. This reflects that the reasons for nurses’ turnover intention have “multiple conjunctural causality” and “equifinality”. “Multiple conjunctural causality” means that an outcome is usually the result of several factors acting together rather than a single factor, and “equifinality” means that the combination of many different factors can lead to the same outcome [22]. Existing research gaps and challenges result in the limited explanatory power of variable‐centered net effects in explaining nurses’ turnover intention, while neglecting the combined effects of different factors [24]. Therefore, it is necessary to explore which combinations (configurations) of the absence of job embeddedness–related factors (dimensions) are more likely to lead to nurses’ turnover intention through a qualitative comparative analysis, as this will enrich and refine existing theories and enhance the effectiveness of practical recommendations aimed at reducing turnover rates. Another strength of qualitative comparative analysis is that it can analyze the asymmetry of nurses’ turnover intention [22]: The reasons that lead to turnover intention and those that prevent it may be different, meaning that eliminating the unfavorable factors that contribute to turnover intention may not necessarily lead to prevent turnover intention altogether. Overall, the objective of this study was to explore how the dimensions of job embeddedness combine to form configurations that influence nurses’ turnover intention or no turnover intention through a fuzzy‐set qualitative comparative analysis. This study provides new insights for healthcare institution administrators to understand why nurses develop turnover intention, and these configurations provide managers with guidance for implementing intervention measures. Based on the configuration of variable combinations, managers can customize targeted management strategies according to individual circumstances and clinical realities, thereby reducing nurse turnover rates.

## 3. Methods

### 3.1. Design

This study design was a cross‐sectional survey using a fuzzy‐set qualitative comparative analysis. The reporting of the study adhered to the Strengthening the Reporting of Observational Studies in Epidemiology (STROBE) statement for cross‐sectional studies [25]. This study is part of the project entitled Turnover Intention Among Nurses in the Guangdong‐Hong Kong‐Macao Greater Bay Area (Project DOI: 10.17605/OSF.IO/9JDFK).

Qualitative comparative analysis is a case‐oriented analytical method grounded in set theory and Boolean logic [26]. In qualitative comparative analysis, the independent and dependent variables are referred to as “conditions” and “outcomes,” respectively, and all study samples are treated as cases. Qualitative comparative analysis treats cases as condition configurations, analyzing the set relationships between specific condition configurations and outcomes [24]. These configurations represent multidimensional combinations of various conditions, reflecting their combined effects. By analyzing the necessity and sufficiency relationships between conditions and outcomes, qualitative comparative analysis helps identify which conditions are necessary for the outcome, which condition configurations lead to the outcome, and which condition configurations lead to the nonoccurrence of the outcome [27]. Fuzzy‐set qualitative comparative analysis is a technique within qualitative comparative analysis that allows the degree of membership in a fuzzy set to range continuously distributed between 0 and 1 when the various kinds of conditions and outcomes cannot be easily classified as 0 and 1 (0 means not membership of a set, and 1 means membership of a set) [28].

### 3.2. Setting and Participants

The study was conducted in six medical institutions in Hong Kong from March to June 2023. The convenience sampling method was used to recruit serving nurses to participate in a self‐report questionnaire. The inclusion criteria were (1) being a registered or enrolled nurse with a valid practice certificate issued by the Hong Kong Nursing Council; (2) being a nurse employed in hospitals or nursing homes; and (3) providing informed consent. Nurses who were not on duty during the study period, including those on vacation, sick leave, personal leave, or continuing education, were excluded.

### 3.3. Sample Size

Unlike quantitative analysis, which requires large samples for sufficient power estimation, and qualitative analysis, which is determined by data saturation, the stability of the qualitative comparative analysis is primarily influenced by the sample’s representativeness. The qualitative comparative analysis method can be applied to small sample sizes (e.g., fewer than 15 cases), medium sample sizes (e.g., 15–50 cases), and large sample sizes (e.g., more than 100 cases) [29]. This study finally included 232 cases, which meet the requirements of the qualitative comparative analysis method.

### 3.4. Data Collection

The qualitative comparative analysis method requires sufficient homogeneity in the case population (cases had similar sociocultural backgrounds, which allowed for comparisons) and maximum heterogeneity within the case population (within the cases, both positive and negative outcomes should be included to ensure diversity of cases) [22]. The researchers selected six medical institutions in Hong Kong, including public hospitals, private hospitals, and nursing homes, to ensure that the participants were both representative and diverse. The online questionnaire was administered through Wenjuanxin and QDiaoyan platforms after obtaining the consent of the Association of Hong Kong Nursing Staff and the medical institutions. The Association of Hong Kong Nursing Staff emailed the link and QR code of the questionnaire to nurses to recruit nurses to participate in this study. The first page of the questionnaire described the study’s aim, meaning, potential risks and benefits, and contact details. A total of 239 nurses participated in this study, and after excluding invalid questionnaires (missing data, outliers, and similar responses), 232 valid questionnaires were included, yielding a validity rate of 97.07%.

### 3.5. Measures

#### 3.5.1. Sociodemographic Questionnaire

The sociodemographic questionnaire was developed by researchers that included gender, age, marital status, education level, type of medical institution, monthly salary, and professional title.

#### 3.5.2. Job Embeddedness Scale

The Chinese version of the Job Embeddedness Scale was used to assess nurses’ job embeddedness [30], which has been widely used and validated in nursing populations [19]. The scale was originally developed by Mitchell et al. [6] to explore the reasons for employee retention and has six dimensions and 37 items: organization fit (7 items), community fit (5 items), organization sacrifice (9 items), community sacrifice (3 items), organization link (7 items), and community link (6 items). Items 1 through 24 use a 5‐point Likert scale (1 = *strongly disagree*, 5 = *strongly agree*); items 25 through 37 are single‐choice questions standardized to Likert scale scores based on the strength of the hypothesized causal relationship. For example, for the item “What is your current marital status?” 1 = “*unmarried*,” 2 = “*divorced without children*,” 3 = “*married without children*” and “*other*,” 4 = “*divorced with children*,” and 5 = “*married with children*.” The total score ranges from 37 to 185, with higher scores indicating higher levels of job embeddedness, and Cronbach’s alpha coefficient for this scale in this study was 0.871.

#### 3.5.3. Turnover Intention

Turnover intention was assessed using a single‐item measure [31], which has been well established as both valid and simple in previous studies [32, 33]. This measure asked participants how often they had seriously considered quitting their job: “In the past year, how often have you thought about leaving your current organization/department?” Response options were: “*never*” = 1, “*annually*” = 2, “*monthly*” = 3, “*weekly*” = 4, and “*daily*” = 5. A higher score indicated a stronger intention to leave, with a score of 1 representing no turnover intention.

### 3.6. Ethical Considerations

This study was approved by the Institutional Review Board of the Academic Committee of Macao Polytechnic University (No. RP/AE‐06/2022). Before administering the questionnaire survey, the researcher explained the purpose and significance of the study to potential participants. All participants were informed that their participation was voluntary and that they could withdraw from the study at any time without penalty. Participants were assured that all data collected would be used solely for academic research purposes, that questionnaire responses would remain anonymous, and that no personal information would be disclosed.

### 3.7. Data Analysis

#### 3.7.1. Descriptive Analysis

Data analysis was conducted using *R* (Version 4.4.2) and fsQCA (Version 3.0). Descriptive statistics were used for continuous variables (job embeddedness and turnover intention) and categorical variables (sociodemographic information). For the continuous variables, the statistics reported included mean, standard deviation, maximum, minimum, and percentiles. For the categorical variables, frequencies and percentages were reported.

#### 3.7.2. Common Method Bias

Both the condition and outcome variables in this study were collected using the same method—a self‐report questionnaire. The condition variables were measured using the Job Embeddedness Scale, and the correlations between the variables were relatively strong. Therefore, the potential effect of common method bias must be considered. We used Harman’s single‐factor test to test for common method bias. For the nonrotated factor analysis, the test included all items related to sociodemographic information, job embeddedness, and turnover intention. If the first factor accounted for less than 50% of the variance [34], the study was considered to have no significant common method bias.

#### 3.7.3. Qualitative Comparative Analysis

1. Calibration: Calibration refers to adjusting measurements to match or confirm known standards. These standards ensure that measurements are consistently interpretable [35]. Calibration in fuzzy‐set qualitative comparative analysis is the process in which set membership scores are assigned to cases [36]. Calibration allows the construction of interval‐ and ratio‐scale source variables as fuzzy‐set [28]. Data calibration can be divided into indirect and direct calibration methods. The indirect method relies on known standard customized membership eigenfunctions and distribution functions, whereas the direct method is achieved through the calibration function in fsQCA 3.0, which is essentially a logistic function [26, 35, 37]. The direct calibration method requires determining thresholds for three anchor points: full membership (fuzzy membership = 0.95), crossover point (fuzzy membership = 0.50), and full nonmembership (fuzzy membership = 0.05). Meaningful calibration criteria should be developed, wherever possible, based on theoretical and substantive knowledge from outside the sample. In the absence of substantive evidence to establish calibration, anchors are typically identified based on the sample’s distribution characteristics [35, 38, 39]. Existing studies typically use quartiles (P75, P50, and P25), specific percentiles (P90, P50, and P10), and means and standard deviations (M + SD, M, and M ‐ SD) as calibration anchors based on the sample distribution characteristics. Since it is difficult to analyze cases in the fuzzy set with membership scores exactly equal to 0.5, a constant of 0.001 was added to the membership scores of all cases less than 1. This adjustment ensured that all cases were retained in the fuzzy‐set analysis. Furthermore, this treatment did not affect the results of the regression analysis conducted in the post hoc analysis [27].
2. Necessity analysis: Necessity analysis examines the extent to which the set of outcomes is a subset of the set of conditions. Specifically, a condition is considered necessary for an outcome if it always occurs whenever that outcome exists [22]. Identifying necessary conditions before conducting a sufficiency analysis helps in making appropriate assumptions about the logical remainders in the Boolean minimization process [40]. The key indicator of necessity conditions is consistency, which is typically recognized as a minimum value of 0.9 [36].
3. Sufficiency analysis: Sufficiency analysis examines whether the configurations formed by different conditions are sufficient for the outcome. First, a truth table was constructed using fsQCA 3.0 to determine the appropriate case frequency, raw consistency, and proportional reduction in inconsistency (PRI) consistency. Case frequency thresholds typically involve trade‐offs based on case size [22], with 80% of cases usually retained [41]. Conditional configurations with fewer cases than the threshold are considered logical remainders. Raw consistency is the probability that a conditional configuration can decisively explain the outcome. Conditional configurations with raw consistency scores greater than or equal to the threshold are considered a subset of the outcome set and are assigned a value of 1. Configurations with raw consistency scores below the threshold are assigned a value of 0. The lowest acceptable threshold is typically set at 0.8 [22]. PRI consistency refers to the degree to which a conditional configuration effectively accounts for the presence of the outcome rather than the absence, thus avoiding the issue of “same cause, different outcome.” The higher the PRI consistency of a conditional configuration, the less likely it is to be part of both the presence and absence of the outcome [36]. Typically, the PRI consistency threshold should be set at ≥ 0.75 [24], which needs to be set according to the truth table, and ≥ 0.60 is also acceptable [42].

Second, to further complete the truth table, it is necessary to address contradictory configurations and logical remainders. Contradictory configurations refer to cases where the same conditional configurations simultaneously lead to the presence and absence of the outcome. In this study, contradictory configurations are identified and excluded by setting thresholds for raw and PRI consistency, which helps exclude configurations that cannot effectively explain the outcome or lead to contradictions. In addition, fsQCA 3.0 uses the Quine–McCluskey minimization algorithm to simplify complex condition configurations automatically, eliminating contradictory configurations [22]. Logical remainders refer to condition configurations that are theoretically possible but lack corresponding instances in the actual case. In this study, logical remainders are identified by setting thresholds for case frequency and raw consistency, and condition configurations that do not meet the threshold are treated as logical remainders. During the minimization process, fsQCA 3.0 simplifies the truth table by merging similar condition combinations. This process reduces logical remainders and generates simplified logical formulas that cover all practical cases as much as possible [22].

Third, after completing the truth table, fsQCA 3.0 analyzes three solutions: the complex solution excluding logical remainders, the parsimonious solution including logical remainders, and the intermediate solution including only those logical remainders consistent with theoretical or practical considerations [22]. The intermediate solution is considered the preferred solution in qualitative comparative analysis research reports [36]. This study makes no additional assumptions about the logical remainder; therefore, fsQCA 3.0 delivers the same intermediate and complex solutions [43].

Fourth, we analyzed the condition configurations associated with the absence of turnover intention to examine the asymmetry of turnover intention. By comparing the configurations of job embeddedness–related conditions across both outcomes (presence and absence of turnover intention), we further explored which condition configurations lead to nurses’ turnover intention and no turnover intention.

Finally, following Fiss’s [27] presentation format, if a condition appears in both the intermediate and parsimonious solutions, it is considered a core condition, indicating that it significantly impacts the outcome. If a condition appears only in the intermediate solution, it is considered a peripheral condition, suggesting that it plays a contributory role to the outcome.

#### 3.7.4. Robustness Check

When performing fuzzy‐set qualitative comparative analysis, researchers face a considerable margin of discretion in setting parameters that can influence the results. Therefore, it is necessary to conduct robustness checks based on the set‐theoretic method. This study achieves robustness checks by adjusting the calibration method and altering the case frequency, raw consistency, and PRI consistency thresholds. The two dimensions for determining robustness are the differences in fit parameters and the set‐relational status of the different formulas. If the differences in solution consistency and coverage are small enough to lead to meaningful and different substantive interpretations, the results can be considered robust. Furthermore, the results can be interpreted as robust if there is a clear subset relationship between different solution terms [36].

#### 3.7.5. Predictive Validity Analysis

Predictive validity analysis assesses the ability of the configured model to predict the outcome variable across different datasets [44]. The original sample was randomly divided into two subsamples: the modeling sample (Subsample 1) and the holdout sample (Subsample 2). Fuzzy‐set qualitative comparative analysis was performed on the subsamples using the same frequency and consistency thresholds as in the main analysis to compare whether the results were similar between the subsamples and the main analysis. A test was then run on Subsample 2 using the configuration model generated for Subsample 1 to determine if it achieved consistency and coverage similar to those of Subsample 1 [45].

#### 3.7.6. Post Hoc Analysis

The qualitative comparative analysis method explores the set relationship between condition and outcome variables and makes causal inferences, not associations. Therefore, using the results from fuzzy‐set qualitative comparative analysis as input variables for statistical analysis can help quantify the fuzzy‐set qualitative comparative analysis outcomes and assess the representativeness of the overall fuzzy‐set qualitative comparative analysis solutions [46, 47]. This study uses ordered logit regression to examine the configured membership of the fuzzy‐set qualitative comparative analysis results and the quantitative associations between the outcome variables. The minimum value of the case condition variables was used as the configuration membership, which served as the independent variable. An ordered logit regression was performed for each independent variable, with turnover intention serving as the dependent variable. Given the variations in turnover intention among different types of nurses, this analysis includes gender, age, marital status, education level, type of medical institution, monthly salary, and professional title as covariates to reduce the impact of confounding and enhance the accuracy of the results.

## 4. Results

### 4.1. Case Characteristics

This study included 232 nurses, of whom 152 (65.5%) were female and 80 (34.5%) were male. Regarding age distribution, 37 nurses (15.9%) were aged ≤ 30 years, 87 (37.5%) were aged 31–40 years, 53 (22.8%) were aged 41–50 years, and 55 (23.7%) were aged ≥ 51 years. More than half of the nurses were married (57.3%), and the majority (87.5%) had a bachelor’s degree or higher. The detailed distribution is shown in Table 2.

**TABLE 2:** Case characteristics.

Variables	*N* (%)
*Gender*	
Male	80 (34.5)
Female	152 (65.5)

*Age*	
≤ 30	37 (15.9)
31–40	87 (37.5)
41–50	53 (22.8)
≥ 51	55 (23.7)

*Marital status*	
Single	88 (37.9)
Married	133 (57.3)
Divorced	11 (4.7)

*Education level*	
Junior college and below	29 (12.5)
Undergraduate	89 (38.4)
Postgraduate and above	114 (49.1)

*Type of medical institution*	
Public hospital	139 (59.9)
Private hospital	29 (12.5)
Nursing home	64 (27.6)

*Monthly salaries (HKD)*	
≤ 40,000	43 (18.5)
40,001–50,000	45 (19.4)
50,001–60,000	56 (24.1)
> 60,000	88 (37.9)

*Professional title*	
Enrolled nurse	19 (8.2)
Registered nurse	108 (46.6)
Registered nurse (specialist)	25 (10.8)
Advanced practice nurse	55 (23.7)
Ward manager/associate nurse consultant	14 (6.0)
Departmental operations manager/nurse consultant	8 (3.4)
General manager of nursing/house director	3 (1.3)

### 4.2. Common Method Bias Test

The results of Harman’s single factor test indicate that 11 factors have eigenvalues greater than 1. The first factor accounts for 22.364% of the variance, which is below the critical threshold of 50% [34]. Therefore, no significant common method bias is present in this study.

### 4.3. Descriptive Statistics and Calibration Values

Table 3 shows descriptive statistics for the main variables, and Supporting Information 1 provides the distribution of the conditional variables. Given the concentrated distribution of the condition variables, using quartiles as anchor points may lead to lower calibration accuracy and insufficient variability. Therefore, in this study, the 90th, 50th, and 10th percentiles were used as anchor points for the condition variables. For the outcome variable (turnover intention), based on the existing criteria of empirical research, “1 point” indicates that participants have no turnover intention at all, “5 points” is considered a strong turnover intention, and “2 points” represents the beginning of turnover intention [31]. Therefore, in calibration, “5 points” is regarded as full membership, “2 points” is regarded as a crossover point, and “1 point” is regarded as full nonmembership.

**TABLE 3:** Descriptive analysis of condition and outcome variables.

Values	Condition variables						Outcome variable
	Organization fit	Community fit	Organization sacrifice	Community sacrifice	Organization link	Community link	Turnover intention
M	23.14	18.53	27.73	9.99	23.08	19.04	2.37
SD	5.27	3.86	5.46	2.09	5.28	5.20	1.36
Min	7.00	5.00	9.00	3.00	11.00	6.00	1.00
Max	35.00	25.00	45.00	15.00	35.00	30.00	5.00
P10	16.30	14.00	20.30	7.00	15.30	12.00	1.00
P25	21.00	16.00	25.00	9.00	19.00	16.00	1.00
P50	23.00	19.00	28.00	10.00	23.50	19.00	2.00
P75	27.00	20.00	31.00	11.00	27.00	23.00	3.00
P90	29.00	24.00	34.00	12.00	30.00	26.00	5.00

*Note:* M, mean; SD, standard deviation; Min, minimum; Max, maximum; P10, 10th percentile; P25, 25th percentile; P50, 50th percentile; P75, 75th percentile; P90, 90th percentile.

### 4.4. Necessity Analysis

Condition variables with a consistency greater than 0.9 with the outcome variable are considered necessary conditions. According to the results of the necessity analysis, job embeddedness–related conditions were not necessary conditions for the presence or absence of turnover intention among nurses. The consistency between these conditions and outcomes was below 0.9, as shown in Table 4.

**TABLE 4:** Necessity analysis.

Conditions	Turnover intention		∼Turnover intention	
	Consistency	Coverage	Consistency	Coverage
OF	0.566	0.502	0.767	0.737
∼OF	0.704	0.736	0.482	0.546
CF	0.603	0.604	0.636	0.691
∼CF	0.691	0.637	0.636	0.634
OS	0.576	0.531	0.745	0.745
∼OS	0.723	0.724	0.531	0.576
CS	0.626	0.567	0.689	0.671
∼CS	0.634	0.653	0.551	0.614
OL	0.598	0.570	0.643	0.663
∼OL	0.647	0.626	0.583	0.611
CL	0.596	0.567	0.675	0.697
∼CL	0.682	0.660	0.581	0.609

*Note:* OF, organization fit; CF, community fit; “∼” means absence and Boolean logic “not.”

Abbreviations: CL = community link, CS = community sacrifice, OL = organization link, OS = organization sacrifice.

### 4.5. Sufficiency Analysis

The calibrated data were used to construct the truth table. This study set the case frequency threshold at 3 and the raw consistency threshold at 0.80, retaining a total of 32 configurations consisting of 197 cases. To retain a sufficient number of configurations as part of the solution set, the PRI consistency threshold was set at 0.60 for the truth table with turnover intention as the outcome variable and 0.65 for the truth table with the absence of turnover intention as the outcome. The truth tables are detailed in Supporting Information 2.

The sufficiency analysis results showed that the overall solution consistencies and the individual solution consistencies were both greater than or close to 0.80 [29], indicating strong correlations between the conditional configurations and the outcomes, as well as strong explanatory power for the outcomes. Five conditional configurations led to nurses’ turnover intention, with an overall solution coverage of 0.525, indicating that these five configurations accounted for 52.5% of turnover intention. According to the definitions of qualitative comparative analysis, all conditions and outcomes can be represented by two states: presence and absence. In this framework, core conditions appear in both the parsimonious and intermediate solutions. These conditions play a critical role in forming configurations and resulting outcomes. In contrast, peripheral conditions appear only in the intermediate solution, exerting a supplementary rather than decisive effect on the outcome. The configurations were grouped based on core conditions and named according to their characteristics, resulting in three groups of conditional configurations that generated nurses’ turnover intention: (1) the on‐the‐job embeddedness absence group (Solution 1a, Solution 1b, and Solution 1c); (2) the off‐the‐job link group (Solution 2); and (3) the on‐ and off‐the‐job embeddedness conflict group (Solution 3).

Meanwhile, the study also analyzed six configurations that contribute to nurses’ no turnover intention. These six configurations explained a total of 66.8% of the factors that kept nurses from developing turnover intention. These configurations were grouped into the following three categories that prevented nurses from developing turnover intention: (1) the fitting embeddedness group (Solution 4a and Solution 4b); (2) the on‐the‐job fit–sacrifice group (Solution 5a, Solution 5b, and Solution 5c); and (3) the link–sacrifice group (Solution 6). Detailed information on the conditional configurations is shown in Tables 5 and 6.

**TABLE 5:** Results of the sufficiency analysis.

Solutions	Turnover intention	∼Turnover intention
Parsimonious solution	∼OF^∗^∼OS^∗^∼OL	OF^∗^CF
	∼CF^∗^∼OS^∗^CL	OF^∗^OS
	∼OF^∗^CF^∗^∼OS^∗^∼CL	OS^∗^OL

Complex (intermediate) solution	∼OF^∗^∼CF^∗^∼OS^∗^∼OL	OF^∗^CF^∗^CS^∗^∼OL
	∼OF^∗^∼OS^∗^CS^∗^∼OL^∗^CL	OF^∗^CF^∗^∼OS^∗^OL^∗^CL
	∼OF^∗^∼OS^∗^∼CS^∗^∼OL^∗^∼CL	OF^∗^OS^∗^CS
	∼OF^∗^∼CF^∗^∼OS^∗^∼CS^∗^CL	OF^∗^∼CF^∗^OS^∗^∼OL^∗^∼CL
	∼OF^∗^CF^∗^∼OS^∗^OL^∗^∼CL	OF^∗^∼CF^∗^OS^∗^OL^∗^CL
		OS^∗^CS^∗^OL^∗^CL

*Note:* OF, organization fit; CF, community fit. “^∗^” means Boolean logic “and.” “∼” means absence and Boolean logic “not.”

Abbreviations: OS = organization sacrifice, CS = community sacrifice, OL = organization link, CL = community link.

**TABLE 6:** Configurations for achieving turnover intention.

Conditions	Turnover intention					∼Turnover intention					
	S1a	S1b	S1c	S2	S3	S4a	S4b	S5a	S5b	S5c	S6
Organization fit	▲	▲	▲	△	▲	●	●	●	●	●	
Community fit	△			▲	●	●	●		△	△	
Organization sacrifice	▲	▲	▲	▲	▲		△	●	●	●	●
Community sacrifice		○	△	△		○		○			○
Organization link	▲	▲	▲		○	△	○		△	○	●
Community link		○	△	●	▲		○		△	○	○
Raw coverage	0.377	0.224	0.236	0.300	0.300	0.347	0.207	0.552	0.267	0.276	0.381
Unique coverage	0.016	0.044	0.012	0.050	0.015	0.021	0.016	0.050	0.023	0.023	0.021
Consistency	0.861	0.880	0.882	0.864	0.865	0.885	0.902	0.827	0.891	0.905	0.899
Solution coverage	0.525					0.668					
Solution consistency	0.829					0.798					

*Note:* S, solution; ● and ○ indicate the presence of the condition; ▲ and △ indicate the absence of the condition; ● and ▲ indicate the core condition; ○ and △ indicate the peripheral condition; blank spaces indicate that the condition is indifferent.

### 4.6. Robustness Check

This study performed four different robustness checks: (1) setting the mean and standard deviation as anchors for data calibration; (2) setting the frequency threshold in the truth table to 2; (3) setting the raw consistency threshold to 0.90; and (4) setting the PRI consistency threshold to 0.70. The fuzzy‐set qualitative comparative analysis was performed after adjusting these parameters separately while keeping other settings constant. The results of the robustness checks showed that the differences in solution coverage and solution consistency were within acceptable limits, with only minor variations observed compared to the main analysis. The results of the robustness checks are detailed in Supporting Information 3. Furthermore, the configurations from the main analysis are either the same as or a subset of those obtained from the robustness checks. Therefore, the results of this study can be considered robust. Table 7 summarizes the relationships between all the robustness checks and the solution terms of the main analysis.

**TABLE 7:** Robustness check summary.

Solution	Configurations identified in main analysis										
	S1a	S1b	S1c	S2	S3	S4a	S4b	S5a	S5b	S5c	S6
Calibration (M + SD, M, M‐SD)	(√)	(√)	√		√			(√)	√	√	(√)
Frequency = 2		(√)	(√)	√	(√)	√	(√)	√	(√)	(√)	√
Consistency > 0.9	(√)		(√)			√		√	(√)		(√)
PRI > 0.7	(√)	(√)	(√)	(√)				(√)		√	(√)

*Note:* M, mean; SD, standard deviation; S, solution; (√) is placed in cases where the same configuration occurs. (√) is placed where the configuration is comparable and is a subset or superset of the main analysis.

### 4.7. Predictive Validity Analysis

The predictive validity analysis showed that the configurations obtained from the subsample analyses were similar to those obtained from the main analysis, as shown in Table 8. The configurations in Subsample 1 were tested using the holdout sample (Subsample 2). As shown in the XY plots in Figure 1, all configurations achieved similar consistency and raw coverage across the different subsamples, demonstrating the ability of the configuration model to predict the nurses’ turnover intention with different datasets.

**TABLE 8:** Configuration analysis of the subsamples.

Sample set	Outcome	Configurations	Raw coverage	Unique coverage	Consistency	Solution coverage	Solution consistency	Relationship with full‐sample configurations
Subsample 1	Turnover intention	∼OF^∗^∼CF^∗^∼OS^∗^∼CS^∗^∼OL	0.319	0.070	0.826	0.451	0.862	Subset of S1a
		∼OF^∗^∼CF^∗^∼OS^∗^∼CS^∗^CL	0.280	0.049	0.567			Same as S2
		∼OF^∗^CF^∗^∼OS^∗^OL^∗^∼CL	0.261	0.089	0.851			Same as S3
	∼Turnover intention	OF^∗^OS^∗^CS^∗^∼OL	0.385	0.020	0.845	0.563	0.881	Same as S4a
		OF^∗^CF^∗^OS^∗^CS	0.458	0.028	0.863			Subset of S5a
		OF^∗^OS^∗^CS^∗^CL	0.444	0.035	0.886			Subset of S5a

Subsample 2	Turnover intention	∼OF^∗^∼CF^∗^∼OS^∗^∼CS^∗^∼OL	0.337	0.081	0.890	0.467	0.810	Subset of S1a
		∼OF^∗^∼CF^∗^∼OS^∗^∼CS^∗^CL	0.319	0.063	0.870			Same as S2
		∼OF^∗^CF^∗^∼OS^∗^CS^∗^∼OL^∗^CL	0.198	0.051	0.917			Subset of S1b
	∼Turnover intention	OF^∗^CF^∗^OS^∗^CS^∗^∼OL	0.316	0.025	0.913	0.551	0.834	Subset of S4a
		OF^∗^CF^∗^OS^∗^CS^∗^CL	0.421	0.107	0.935			Subset of S5a
		OF^∗^∼CF^∗^OS^∗^∼CS^∗^∼OL^∗^∼CL	0.215	0.047	0.924			Subset of S5b
		OF^∗^∼CF^∗^OS^∗^∼CS^∗^OL^∗^CL	0.231	0.036	0.892			Subset of S5c

*Note:* OF, organization fit; CF, community fit; S, solution. “^∗^” means Boolean logic “and.” “∼” means absence and Boolean logic “not.”

Abbreviations: CL = community link, CS = community sacrifice, OL = organization link, OS = organization sacrifice.

FIGURE 1XY scatter plot of complex configurations in different subsamples.

**Figure:**
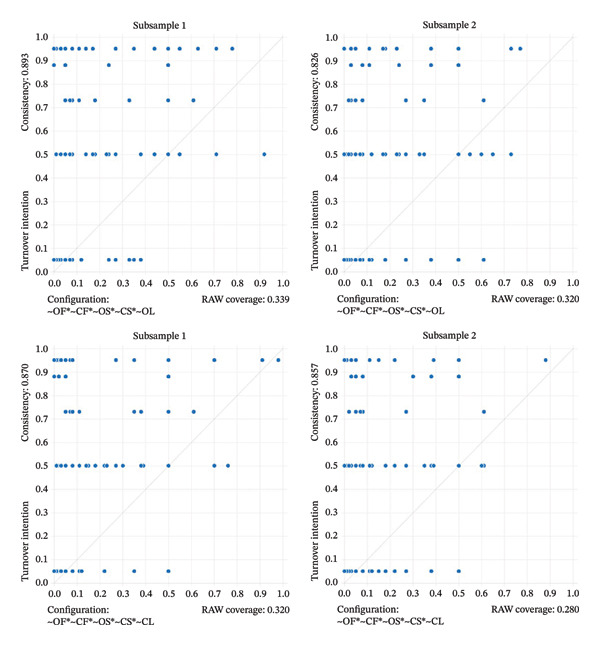

**Figure:**
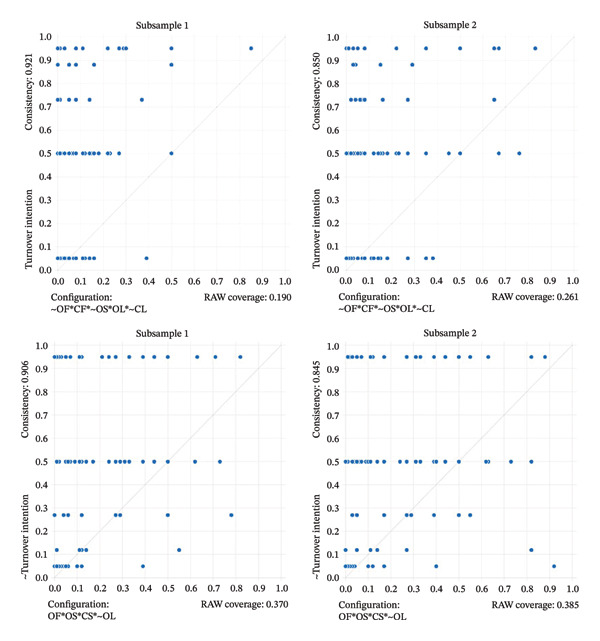

**Figure:**
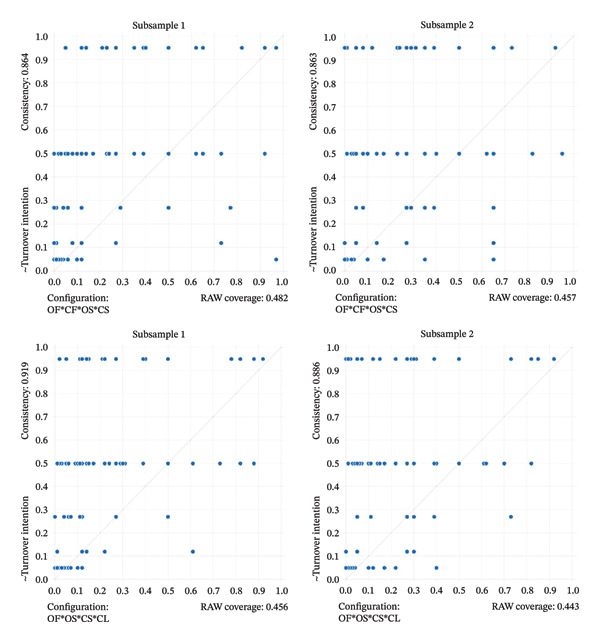

### 4.8. Post Hoc Analysis

The results of the post hoc analysis (Table 9) show that configurations Solution 1a, Solution 1c, Solution 2, and Solution 3 are all significantly positively associated with turnover intention (Solution 1a: *B* = 3.098, *p* = 0.007; Solution 1c: *B* = 2.031, *p* = 0.004; Solution 2: *B* = 2.114, *p* = 0.002; Solution 3: *B* = 2.940, *p* < 0.001), while configurations Solution 4a, Solution 4b, Solution 5a, Solution 5b, Solution 5c, and Solution 6 are significantly negatively associated with turnover intention (Solution 4a: *B* = −1.648, *p* = 0.010; Solution 4b: *B* = −1.930, *p* = 0.044; Solution 5a: *B* = −2.546, *p* < 0.001; Solution 5b: *B* = −1.672, *p* = 0.030; Solution 5c: *B* = −2.797, *p* < 0.001; Solution 6: *B* = −3.302, *p* < 0.001). Given that the post hoc analysis complements the main analysis, and considering that a *p* value of less than 0.1 may indicate a meaningful trend, the positive association between Solution 1b and turnover intention is considered marginally significant (Solution 1b: *B* = 1.729, *p* = 0.077). Overall, these results are largely consistent with those obtained through the fuzzy‐set qualitative comparative analysis.

**TABLE 9:** Results of the ordered logit regression analysis for turnover intention.

Variables	Independent variable: turnover intention			
	B	SE	*t*	*p*
S1a	3.098	1.165	2.659	0.007
S1b	1.729	0.981	1.761	0.077
S1c	2.031	0.708	2.868	0.004
S2	2.114	0.689	3.067	0.002
S3	2.940	0.084	3.495	< 0.001
S4a	−1.648	0.651	−2.529	0.010
S4b	−1.930	0.972	−1.985	0.044
S5a	−2.546	0.528	−4.823	< 0.001
S5b	−1.671	0.782	−2.138	0.030
S5c	−2.797	0.816	−3.426	< 0.001
S6	−3.302	0.675	−4.895	< 0.001

*Note:* OF, organization fit; CF, community fit; “∼” means absence and Boolean logic “not.”

Abbreviations: CL = community link, CS = community sacrifice, OL = organization link, OS = organization sacrifice.

## 5. Discussion

This is, to the best of our knowledge, the first study to apply the qualitative comparative analysis method to examine how multiple constructs of job embeddedness combine to form configurations that contribute to or prevent nurses from developing turnover intention. The results of the necessary analysis showed that no one job embeddedness–related factor was alone a necessary condition for either contributing to or deterring nurses’ turnover intention. The sufficiency analysis revealed five configurations that contribute to nurses’ turnover intention and six configurations that are related to nurses’ no turnover intention. These configurations had acceptable robustness and predictive validity. This study revealed the intrinsic mechanisms by which the absence of job embeddedness contributes to nurses’ turnover intention. These condition configurations, which reflect the combined effects of multiple variables on nurses’ turnover intention, provide managers with new insights into the reasons why nurses leave. It highlights that turnover intention is not caused by any single job embeddedness–related factor but rather by the combined effect of specific factors. Meanwhile, these findings provide a theoretical foundation for future configuration‐based management strategies. Traditional management strategies are often constrained by real‐world conditions, making them unsuitable for all situations and nursing staff. In contrast, this study highlights that nursing management interventions designed to facilitate the formation of optimal condition configurations may be more flexible and effective.

### 5.1. Configurations Contributing to Nurses’ Turnover Intention

Looking across all configurations that contribute to nurses’ turnover intention, the absence of organization sacrifice emerges as a core condition across all configurations, and the absence of organization fit is a core condition in all configurations except Solution 2. Consistent with the findings of the Wang et al. [19] meta‐analysis, among the six dimensions of job embeddedness, organization sacrifice (−0.505) and organization fit (−0.488) have the strongest correlations with turnover intention. This highlights the centrality of these two conditions: Administrators should prioritize addressing these issues to prevent higher turnover intention among nurses. Specifically, the simultaneous mismatch between job content and expectations, along with insufficient benefits, may be the primary factors contributing to nurses’ turnover intention. Nursing managers should engage in regular communication with nurses to identify gaps between their job expectations and actual task assignments, with particular attention to nurses who receive lower levels of benefits and those with higher educational qualifications. Through flexible adjustment of job responsibilities and task allocation, efforts should be made to better align nurses’ work content with their individual expectations. In addition, for nurses facing high workloads and newly graduated nurses undergoing transitional adjustment challenges, administrators should provide competitive benefit packages through supportive policies such as clear career advancement pathways and flexible work arrangements.

Based on the core conditions, we categorized the configurations contributing to nurses’ turnover intention into three groups. The first group is the on‐the‐job embeddedness absence group. The central feature of this group is the absence of organization embeddedness, meaning that nurses simultaneously lack organization fit, organization sacrifice, and organization link. This group highlights the impact of work‐related forces within the organization on nurses’ turnover intention. Previous studies [48, 49] also suggest that organizational factors, such as working conditions and the workplace environment, have a broad and complex influence on both turnover intention and retention among nurses. This group can be further divided into three different configurations. In addition to the absence of organization embeddedness, Solution 1a is accompanied by the absence of community fit; Solution 1b is accompanied by the simultaneous presence of community sacrifice and link; and Solution 1c is accompanied by the simultaneous absence of community sacrifice and link, which can be considered the total absence of job embeddedness, with the highest consistency (0.865). Will community embeddedness help keep nurses in their jobs if they are not strongly embedded in the organization? The answer is no. Comparing these configurations shows that when organization embeddedness is absent, the presence or absence of community embeddedness (especially sacrifice and link) at the same time leads to turnover intention. This may contradict the traditional hypothesis that promoting community embeddedness reduces nurses’ turnover intention [6]. The results of this study suggest that when nurses lack organization embeddedness, higher community embeddedness further contributes to their turnover intention. This could be interpreted to mean that when organizational factors are insufficient to retain nurses (e.g., low income, poor work environment, etc.), nurses may leave their current jobs due to the need to take on more family responsibilities [50]. Therefore, this finding suggests that once new nurses are employed, helping them develop a strong relationship with the organization/job should be a priority for retention, followed by retaining nurses through measures outside of work. Another finding is that community fit, or the combination of community sacrifice–link, played the same role. Based on the scale’s items, it can be assumed that community fit reflected nurses’ subjective liking of their communities, while community link and sacrifice represented objective connections between nurses and their communities. This suggests that when nurses are not embedded in their organization, any lack of subjective or objective relationship with the community creates the most severe configurations of turnover intention.

The off‐the‐job link group is characterized by nurses who have strong connections to their community and family. This means that nurses may have to take on some responsibility for their family, yet they lack the match to their work and family. Meanwhile, leaving their job may not pose a significant cost to their current life. The findings suggest that when nurses lack fit and sacrifice to both their organizations and communities, efforts to strengthen their community link not only fail to reduce turnover intention but may even result in configurations that increase turnover intention. This finding adds to job embeddedness theory, which suggests that any increase in “force” will reduce nurses’ turnover intention, but this study adds that the interplay of multiple factors must be considered and that single‐factor interventions may be counterproductive. This also explains why some studies show a significant positive correlation between nurses’ link and turnover intention [20, 22]. Additionally, due to the global shortage of nurses, more and more countries and regions, such as Hong Kong, are addressing this issue by recruiting expatriate/nonlocal nurses [51–53]. However, expatriate nurses often face significant challenges, including immigration pressures and culture shock, which make retention difficult [52]. Previous studies have identified several factors that can facilitate the retention of expatriate nurses, such as a sense of belonging, recognition of different cultures, favorable economic conditions, and other work‐related factors [51, 54]. This result provides fresh insights by suggesting that administrators and policymakers should prioritize supporting nonlocal nurses in adapting to their work content and environment. This can be achieved by offering adequate benefits, training, and career development opportunities. Once these factors are addressed, efforts to strengthen the connection between nurses and the local community can more effectively enhance nurse retention.

The third group describes a configuration where nurses experience conflicts in on‐ and off‐the‐job embeddedness: Nurses have some years of experience in their current unit and take on a relatively large number of departmental affairs, but they feel mismatched for the position. They prefer the environment and atmosphere of the community where they live over the workplace, but they have few connections to the community. These on‐ and off‐the‐job embeddedness conflicts indirectly reflect the work–family conflicts that nurses face, similar to the study by Yildiz et al. [55]. Given the unique nature of the nursing profession and its critical role in the healthcare system, work–family conflicts have become increasingly complex, and the imbalance between nurses’ work and family roles is a significant contributor to turnover intention [56, 57]. Managers play a critical role in addressing work–family conflicts among nurses. Specifically, they must fully understand the complexity of nursing work and adopt targeted management strategies, such as supportive supervisory behaviors, organizational support, and alternative work arrangements, to reduce both existing and potential conflicts and their negative consequences [55, 58, 59].

### 5.2. Configurations Related to Nurses’ No Turnover Intention

The three groups of configurations demonstrate how job embeddedness–related conditions cooperate and substitute for each other to reduce nurses’ turnover intention. Organization sacrifice is a core condition in all configurations that lead to nurses’ turnover intention. However, configurations that are related to no turnover intention show that when nurses are well fitted and connected to both their current organization and community, turnover intention does not occur, even in the absence of organization sacrifice. Additionally, if organization sacrifice is present, nurses will not have a turnover intention if any of the conditions of the organization fit and organization link are met. The characteristic of the fitting embeddedness group is that it meets the conditions of both organization and community fit, similar to the study by Li et al. [60], who found that new nurses have a strong turnover intention, which is significantly and negatively associated with person–organization fit. The support from ward managers and colleagues plays a key role in facilitating the fit between nurses and the work environment, helping new nurses reduce job stress and settle into their roles as quickly as possible [61]. Meanwhile, Solution 4b provides a configuration in which the absence of organization sacrifice may prevent nurses from developing turnover intention. Generally, nurses with limited professional development may have lower levels of organization sacrifice and will seek jobs that are more conducive to their development. This configuration suggests that these nurses can be helped to connect with the organization through several strategies, including career planning support, good orientation, development opportunities, and organizational/psychological empowerment to take on some responsibilities within the unit. These strategies can help nurses form nonturnover configurations, thereby reducing the turnover rate of these nurses.

The configuration that satisfies both the organization fit and organization sacrifice conditions has the highest raw coverage (Solution 5a), meaning that this configuration explains 55.2% of the cases of nurses with no turnover intention, similar to the results found for the turnover intention configurations. The group of configurations also indicates that when core conditions are met but community fit is lacking, neither the presence nor absence of the organization link and the community link will trigger turnover intention. Nurses with both links may be considered experienced nurses, while the absence of both links suggests they may be new nurses. Both lacked community fit and may be less likely to take on too many family responsibilities. These two configurations can represent “rising stars” and “workaholics” among nurses, respectively, whose lives are centered on their work and are well adapted to their current jobs, making them more inclined to stay in their jobs [50]. This group further emphasizes the importance of organization fit and organization sacrifice in reducing nurses’ turnover intention.

Compared to the on‐the‐job fit–sacrifice group of configurations, “link” replaces “fit” in the link–sacrifice group of configurations. Satisfying both sacrifice and link factors also prevents nurses from developing turnover intention, regardless of whether they are a good fit for their jobs. Based on the motivation–hygiene theory, which divides organizational factors into motivational and hygiene factors [63], these “link” and “sacrifice” dimensions of job embeddedness theory may be closer to hygiene factors, which primarily affect employees’ job dissatisfaction because they are linked to interpersonal relationships and salary compensation. Consequently, if these hygiene factors are met, nurses’ dissatisfaction and turnover intentions can be alleviated while productivity increases. Moreover, this configuration emphasizes the importance of work in an individual’s life rather than focusing solely on the content of the work. It suggests that managers need to consider not only the fit of job content but also the impact of work on factors outside of work, such as increasing nurses’ life satisfaction and promoting work–life balance [64].

### 5.3. Limitations

This study is the first to use the qualitative comparative analysis method to explore how the absence of job embeddedness contributes to nurses’ turnover intention, resulting in some effective configurations. However, the study has several limitations. First, participants were selected from six medical institutions in Hong Kong, and the relatively small sample of nurses in specific cultural contexts may limit the generalizability of the findings to other cultural settings. Therefore, policymakers in countries or regions with different healthcare systems, rural settings, and nurse staffing policies should exercise caution when interpreting this study’s findings. Second, the data collected from cross‐sectional surveys cannot establish a direct causal relationship between the condition and outcome variables. Additionally, qualitative comparative analysis examines the configurations of condition variables within specific groups that lead to particular outcomes. Further research should employ alternative methods to explore why nurses with different characteristics develop turnover intention. Third, both job embeddedness and turnover intention are self‐perceived concepts, which are reasonable for the usage of a self‐reported questionnaire, but this may cause the bias of the data. Finally, although a single‐item measure of turnover intention provides a clear anchor point for data calibration, it is important to note that, as a complex psychological construct, a single‐item measure may not fully capture its multidimensional nature. Moreover, turnover intention does not necessarily translate to actual turnover behavior, and future research should further consider the diversity of turnover behaviors.

## 6. Conclusions

Nurses’ turnover intention is usually the result of a combination of factors. This study used the fuzzy‐set qualitative comparative analysis to explore how the six constructs of job embeddedness combine to influence nurses’ turnover intention. Six groups of configurations resulting in turnover intention and no turnover intention were identified, offering new insights for administrators and policymakers. Specifically, the configuration results provide administrators with a deeper understanding of how multiple factors interact within complex clinical settings to affect nurses’ turnover intention. This knowledge helps administrators develop targeted strategies by optimizing the work environment and organizational structure, balancing work and community roles, and enhancing nurses’ sense of organizational belonging. Meanwhile, when some nurses’ needs cannot be met in a short period, such as a lack of community connection or a lower salary, administrators can promote the formation of the optimal configuration that prevents turnover intention through alternative strategies. This study also highlights that the absence of organization fit and organization sacrifice is a core condition in nearly all configurations, leading to nurses’ turnover intention. Therefore, policymakers and administrators should focus on these two factors, providing timely support to prevent nurses from facing the dual dilemma of job misfit and insufficient work incentives and compensation, which may lead to turnover intention.

## Author Contributions

Xin Wang: conceptualization, data curation, formal analysis, methodology, writing–original draft, and writing–review and editing. Ming Liu: conceptualization, data curation, funding acquisition, methodology, project administration, supervision, writing–original draft, and writing–review and editing. Jun‐E. Zhang and Renli Deng: data curation, investigation, writing–original draft, and writing–review and editing. Mengqi Li: formal analysis, methodology, writing–original draft, and writing–review and editing. Xiaoyan Jin and Hongxia Dai: validation, writing–original draft, and writing–review and editing. Yan Wang: visualization, writing–original draft, and writing–Review and editing. Xin Wang: writing–original draft and writing–review and editing. Angela Yee Man Leung: conceptualization, investigation, methodology, project administration, supervision, writing–original draft, and writing–review and editing.

## Funding

This study was funded by the Academic Research Funding of Macao Polytechnic University (Grant number RP/AE‐06/2022) and the Research Fund (Grant number ZH8M) of The Hong Kong Polytechnic University.

## Conflicts of Interest

The authors declare no conflicts of interest.

## Supporting Information

Additional supporting information can be found online in the Supporting Information section. This study includes Supporting Information 1, 2, and 3. Supporting Information 1 presents the histogram of the condition variables’ distribution. Supporting Information 2 shows truth tables. Supporting Information 3 presents the results of the robustness checking configurations.

## Supporting information


**Supporting Information 1** Histograms of the distribution of the condition variables.

**Supporting Information 2** Truth table.

**Supporting Information 3** Results of robustness checking configurations.

## Data Availability

The data that support the findings of this study are available on request from the corresponding author. The data are not publicly available due to privacy or ethical restrictions.
