# Supplementary material for: How Does the Absence of Job Embeddedness Contribute to Nurses’ Turnover Intention? A Fuzzy‐Set Qualitative Comparative Analysis
Source: J Nurs Manag. 2026 Jun 19;2026:2341935. doi: 10.1155/jonm/2341935 (PMC13282276; doi:10.1155/jonm/2341935)
Supplement: Supplementary file 1 — Supporting Information 1 Histograms of the distribution of the condition variables. [file JONM-2026-2341935-s002.docx]

**Supporting Information 1** Histogram of the distribution of the condition variables


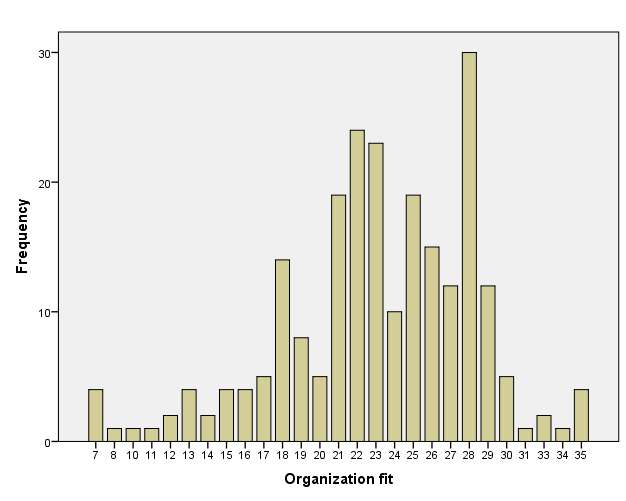


**Figure S1** Histogram of the distribution of organization fit


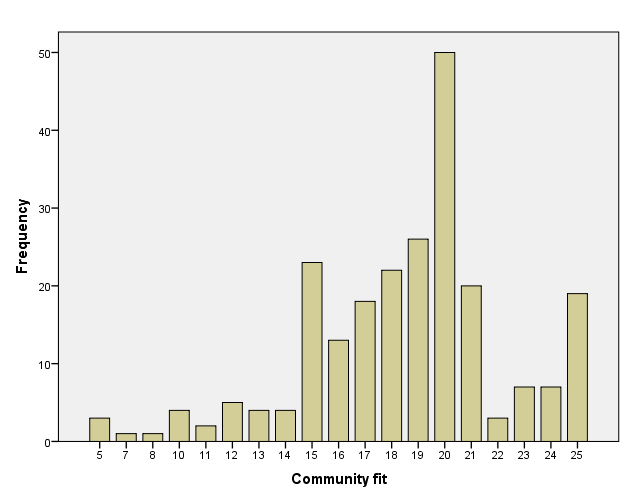


**Figure S2** Histogram of the distribution of community fit


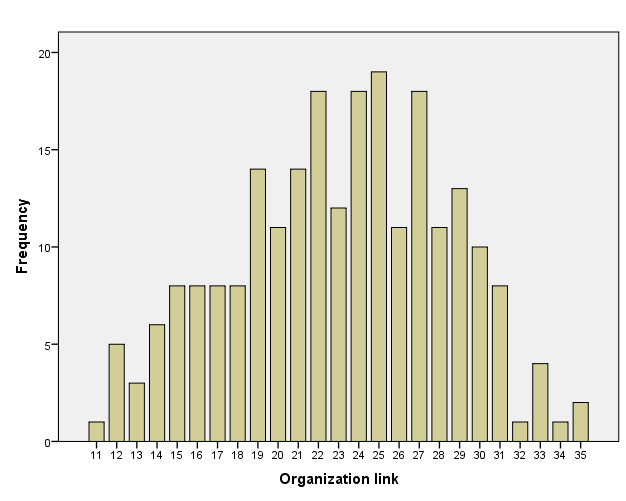


**Figure S3** Histogram of the distribution of organization link


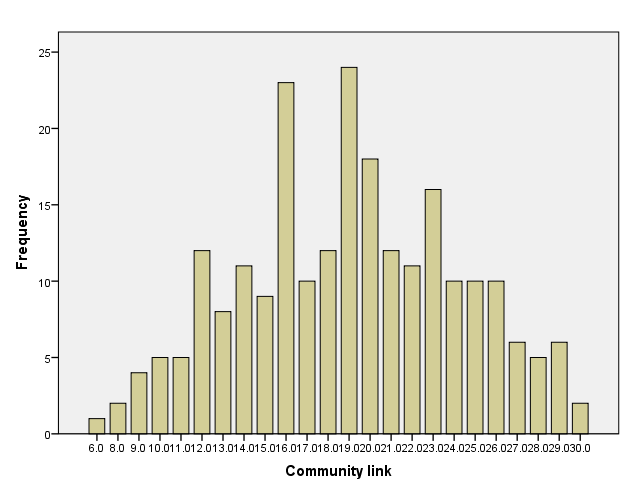


**Figure S4** Histogram of the distribution of community link


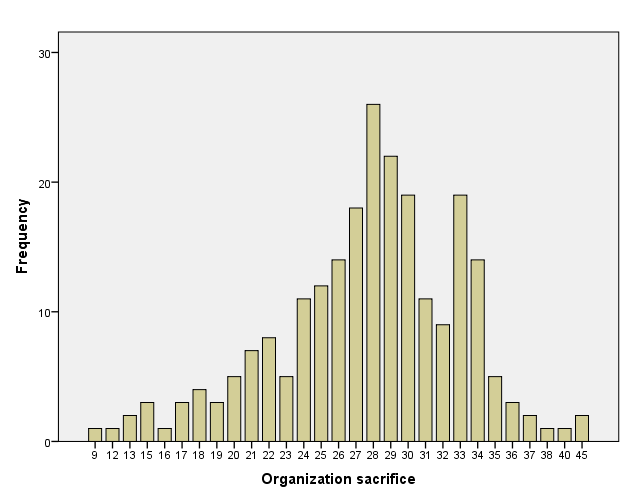


**Figure S5** Histogram of the distribution of organization sacrifice


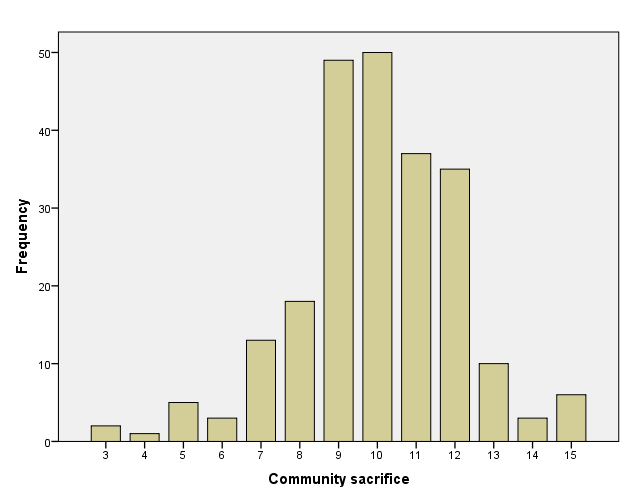


**Figure S6** Histogram of the distribution of community sacrifice
