# Supplementary material for: How Does the Absence of Job Embeddedness Contribute to Nurses’ Turnover Intention? A Fuzzy‐Set Qualitative Comparative Analysis
Source: J Nurs Manag. 2026 Jun 19;2026:2341935. doi: 10.1155/jonm/2341935 (PMC13282276; doi:10.1155/jonm/2341935)
Supplement: Supplementary file 2 — Supporting Information 2 Truth table. [file JONM-2026-2341935-s003.docx]

**Supporting Information 2** Truth table

**Table S1** Truth table for the outcome variable as turnover intention

| OF | CF | OS | CS | OL | CL | number | TI | raw consist. | PRI consist. | SYM consist. |
| --- | --- | --- | --- | --- | --- | --- | --- | --- | --- | --- |
| 1 | 1 | 1 | 1 | 1 | 1 | 29 |  | 0.5872 | 0.076215 | 0.0825316 |
| 1 | 1 | 1 | 1 | 0 | 1 | 15 |  | 0.701528 | 0.171724 | 0.174919 |
| 1 | 1 | 1 | 1 | 0 | 0 | 11 |  | 0.797597 | 0.301952 | 0.313867 |
| 1 | 1 | 1 | 1 | 1 | 0 | 11 |  | 0.75383 | 0.215056 | 0.231663 |
| 0 | 0 | 0 | 0 | 0 | 0 | 10 |  | 0.867684 | 0.624505 | 0.678628 |
| 0 | 0 | 0 | 0 | 0 | 1 | 8 |  | 0.912089 | 0.752703 | 0.756206 |
| 0 | 0 | 0 | 0 | 1 | 1 | 8 |  | 0.852003 | 0.60734 | 0.644306 |
| 1 | 0 | 1 | 0 | 1 | 1 | 8 |  | 0.704221 | 0.246924 | 0.252203 |
| 0 | 0 | 0 | 0 | 1 | 0 | 7 |  | 0.855981 | 0.580948 | 0.601712 |
| 1 | 0 | 1 | 0 | 0 | 0 | 6 |  | 0.793921 | 0.289609 | 0.297873 |
| 0 | 1 | 0 | 1 | 0 | 1 | 6 |  | 0.893826 | 0.629536 | 0.63932 |
| 1 | 0 | 1 | 1 | 0 | 0 | 5 |  | 0.803161 | 0.295072 | 0.30338 |
| 0 | 1 | 1 | 1 | 0 | 0 | 5 |  | 0.862251 | 0.455685 | 0.463726 |
| 1 | 0 | 0 | 0 | 1 | 0 | 5 |  | 0.816803 | 0.36369 | 0.36895 |
| 0 | 1 | 0 | 0 | 1 | 0 | 5 |  | 0.876525 | 0.623646 | 0.66029 |
| 0 | 1 | 0 | 1 | 1 | 0 | 5 |  | 0.890859 | 0.685721 | 0.685721 |
| 1 | 0 | 1 | 1 | 1 | 1 | 5 |  | 0.72895 | 0.137903 | 0.138757 |
| 0 | 1 | 0 | 0 | 0 | 0 | 4 |  | 0.92834 | 0.746451 | 0.763032 |
| 0 | 1 | 1 | 1 | 0 | 1 | 4 |  | 0.820286 | 0.391481 | 0.391481 |
| 1 | 1 | 0 | 1 | 1 | 1 | 4 |  | 0.764081 | 0.186662 | 0.186662 |
| 0 | 0 | 0 | 1 | 0 | 0 | 3 |  | 0.926632 | 0.736652 | 0.736652 |
| 1 | 0 | 0 | 1 | 0 | 0 | 3 |  | 0.885777 | 0.368841 | 0.376612 |
| 1 | 1 | 0 | 1 | 0 | 0 | 3 |  | 0.881473 | 0.286684 | 0.293652 |
| 1 | 0 | 1 | 1 | 1 | 0 | 3 |  | 0.801757 | 0.252596 | 0.252596 |
| 0 | 0 | 0 | 1 | 0 | 1 | 3 |  | 0.907201 | 0.65835 | 0.658349 |
| 1 | 1 | 0 | 1 | 0 | 1 | 3 |  | 0.83486 | 0.229886 | 0.234284 |
| 0 | 0 | 1 | 1 | 0 | 1 | 3 |  | 0.85559 | 0.398367 | 0.398366 |
| 1 | 0 | 1 | 1 | 0 | 1 | 3 |  | 0.788403 | 0.20589 | 0.209232 |
| 1 | 1 | 0 | 0 | 1 | 1 | 3 |  | 0.783128 | 0.326322 | 0.333632 |
| 0 | 1 | 0 | 1 | 1 | 1 | 3 |  | 0.864291 | 0.590131 | 0.590131 |
| 0 | 0 | 1 | 1 | 1 | 1 | 3 |  | 0.820269 | 0.300918 | 0.306155 |
| 0 | 1 | 1 | 1 | 1 | 1 | 3 |  | 0.77259 | 0.272844 | 0.272844 |
| 1 | 0 | 0 | 0 | 0 | 0 | 2 |  | 0.851521 | 0.385897 | 0.397001 |
| 0 | 1 | 1 | 0 | 0 | 0 | 2 |  | 0.899966 | 0.553761 | 0.55376 |
| 1 | 1 | 1 | 0 | 0 | 0 | 2 |  | 0.824602 | 0.258122 | 0.265144 |
| 0 | 1 | 0 | 1 | 0 | 0 | 2 |  | 0.925055 | 0.726154 | 0.731303 |
| 0 | 0 | 1 | 0 | 0 | 1 | 2 |  | 0.876306 | 0.538487 | 0.548058 |
| 1 | 0 | 1 | 0 | 0 | 1 | 2 |  | 0.811925 | 0.319633 | 0.319633 |
| 1 | 0 | 0 | 1 | 0 | 1 | 2 |  | 0.880867 | 0.356659 | 0.356659 |
| 0 | 1 | 0 | 0 | 1 | 1 | 2 |  | 0.849168 | 0.579448 | 0.579448 |
| 0 | 1 | 1 | 0 | 1 | 1 | 2 |  | 0.807077 | 0.372152 | 0.372152 |
| 1 | 1 | 1 | 0 | 1 | 1 | 2 |  | 0.685326 | 0.199148 | 0.199148 |
| 1 | 1 | 0 | 0 | 0 | 0 | 1 |  | 0.89197 | 0.341464 | 0.350997 |
| 0 | 0 | 1 | 0 | 0 | 0 | 1 |  | 0.867445 | 0.511195 | 0.511195 |
| 0 | 0 | 1 | 1 | 0 | 0 | 1 |  | 0.888127 | 0.551724 | 0.551724 |
| 1 | 1 | 0 | 0 | 1 | 0 | 1 |  | 0.821504 | 0.271829 | 0.271829 |
| 1 | 0 | 1 | 0 | 1 | 0 | 1 |  | 0.787553 | 0.278219 | 0.278219 |
| 1 | 1 | 1 | 0 | 1 | 0 | 1 |  | 0.779491 | 0.212675 | 0.212675 |
| 0 | 0 | 0 | 1 | 1 | 0 | 1 |  | 0.897284 | 0.621047 | 0.621046 |
| 0 | 0 | 1 | 1 | 1 | 0 | 1 |  | 0.863185 | 0.40781 | 0.40781 |
| 1 | 0 | 0 | 0 | 0 | 1 | 1 |  | 0.881066 | 0.498881 | 0.498881 |
| 0 | 1 | 0 | 0 | 0 | 1 | 1 |  | 0.899855 | 0.646128 | 0.646128 |
| 0 | 1 | 1 | 0 | 0 | 1 | 1 |  | 0.874905 | 0.49369 | 0.493691 |
| 1 | 1 | 1 | 0 | 0 | 1 | 1 |  | 0.82685 | 0.275439 | 0.275439 |
| 1 | 0 | 0 | 0 | 1 | 1 | 1 |  | 0.811455 | 0.406516 | 0.406517 |
| 0 | 0 | 1 | 0 | 1 | 1 | 1 |  | 0.827275 | 0.449749 | 0.449749 |
| 0 | 0 | 0 | 1 | 1 | 1 | 1 |  | 0.877154 | 0.571348 | 0.571348 |
| 0 | 0 | 1 | 0 | 1 | 0 | 0 |  |  |  |  |
| 0 | 1 | 1 | 0 | 1 | 0 | 0 |  |  |  |  |
| 1 | 0 | 0 | 1 | 1 | 0 | 0 |  |  |  |  |
| 1 | 1 | 0 | 1 | 1 | 0 | 0 |  |  |  |  |
| 0 | 1 | 1 | 1 | 1 | 0 | 0 |  |  |  |  |
| 1 | 1 | 0 | 0 | 0 | 1 | 0 |  |  |  |  |
| 1 | 0 | 0 | 1 | 1 | 1 | 0 |  |  |  |  |
| Note: OF, organization fit; CF, community fit; OS, organization sacrifice; CS, community sacrifice; OL, organization link; CL, community link; "~" mean absence and boolean logic "not". | | | | | | | | | | |

**Table S2** Truth table for the outcome variable as the absence of turnover intention

| OF | CF | OS | CS | OL | CL | number | ~TI | raw consist. | PRI consist. | SYM consist. |
| --- | --- | --- | --- | --- | --- | --- | --- | --- | --- | --- |
| 1 | 1 | 1 | 1 | 1 | 1 | 29 |  | 0.931742 | 0.847249 | 0.917468 |
| 1 | 1 | 1 | 1 | 0 | 1 | 15 |  | 0.931535 | 0.810007 | 0.825081 |
| 1 | 1 | 1 | 1 | 0 | 0 | 11 |  | 0.90144 | 0.660086 | 0.686133 |
| 1 | 1 | 1 | 1 | 1 | 0 | 11 |  | 0.910074 | 0.713258 | 0.768337 |
| 0 | 0 | 0 | 0 | 0 | 0 | 10 |  | 0.751834 | 0.295741 | 0.321373 |
| 0 | 0 | 0 | 0 | 0 | 1 | 8 |  | 0.730776 | 0.242664 | 0.243793 |
| 0 | 0 | 0 | 0 | 1 | 1 | 8 |  | 0.749464 | 0.335286 | 0.355694 |
| 1 | 0 | 1 | 0 | 1 | 1 | 8 |  | 0.894797 | 0.732146 | 0.747796 |
| 0 | 0 | 0 | 0 | 1 | 0 | 7 |  | 0.788481 | 0.384545 | 0.398289 |
| 1 | 0 | 1 | 0 | 0 | 0 | 6 |  | 0.907939 | 0.682647 | 0.702127 |
| 0 | 1 | 0 | 1 | 0 | 1 | 6 |  | 0.815191 | 0.355158 | 0.360679 |
| 1 | 0 | 1 | 1 | 0 | 0 | 5 |  | 0.90996 | 0.677543 | 0.69662 |
| 0 | 1 | 1 | 1 | 0 | 0 | 5 |  | 0.880293 | 0.526974 | 0.536274 |
| 1 | 0 | 0 | 0 | 1 | 0 | 5 |  | 0.891188 | 0.622054 | 0.631051 |
| 0 | 1 | 0 | 0 | 1 | 0 | 5 |  | 0.777185 | 0.320857 | 0.339711 |
| 0 | 1 | 0 | 1 | 1 | 0 | 5 |  | 0.761867 | 0.314279 | 0.314279 |
| 1 | 0 | 1 | 1 | 1 | 1 | 5 |  | 0.954707 | 0.855941 | 0.861242 |
| 0 | 1 | 0 | 0 | 0 | 0 | 4 |  | 0.782892 | 0.231817 | 0.236967 |
| 0 | 1 | 1 | 1 | 0 | 1 | 4 |  | 0.884384 | 0.608518 | 0.608519 |
| 1 | 1 | 0 | 1 | 1 | 1 | 4 |  | 0.945856 | 0.813336 | 0.813336 |
| 0 | 0 | 0 | 1 | 0 | 0 | 3 |  | 0.794772 | 0.263349 | 0.263349 |
| 1 | 0 | 0 | 1 | 0 | 0 | 3 |  | 0.929516 | 0.610526 | 0.623387 |
| 1 | 1 | 0 | 1 | 0 | 0 | 3 |  | 0.948421 | 0.689588 | 0.706349 |
| 1 | 0 | 1 | 1 | 1 | 0 | 3 |  | 0.933001 | 0.747403 | 0.747403 |
| 0 | 0 | 0 | 1 | 0 | 1 | 3 |  | 0.821178 | 0.34165 | 0.34165 |
| 1 | 1 | 0 | 1 | 0 | 1 | 3 |  | 0.946678 | 0.75134 | 0.765716 |
| 0 | 0 | 1 | 1 | 0 | 1 | 3 |  | 0.90438 | 0.601634 | 0.601634 |
| 1 | 0 | 1 | 1 | 0 | 1 | 3 |  | 0.940883 | 0.778138 | 0.790768 |
| 1 | 1 | 0 | 0 | 1 | 1 | 3 |  | 0.887896 | 0.651767 | 0.666368 |
| 0 | 1 | 0 | 1 | 1 | 1 | 3 |  | 0.804606 | 0.409869 | 0.409869 |
| 0 | 0 | 1 | 1 | 1 | 1 | 3 |  | 0.918238 | 0.681976 | 0.693845 |
| 0 | 1 | 1 | 1 | 1 | 1 | 3 |  | 0.914671 | 0.727156 | 0.727156 |
| 1 | 0 | 0 | 0 | 0 | 0 | 2 |  | 0.899935 | 0.586135 | 0.602999 |
| 0 | 1 | 1 | 0 | 0 | 0 | 2 |  | 0.875863 | 0.446239 | 0.446239 |
| 1 | 1 | 1 | 0 | 0 | 0 | 2 |  | 0.932713 | 0.715395 | 0.734856 |
| 0 | 1 | 0 | 1 | 0 | 0 | 2 |  | 0.799341 | 0.266804 | 0.268697 |
| 0 | 0 | 1 | 0 | 0 | 1 | 2 |  | 0.850994 | 0.444049 | 0.451942 |
| 1 | 0 | 1 | 0 | 0 | 1 | 2 |  | 0.911643 | 0.680366 | 0.680366 |
| 1 | 0 | 0 | 1 | 0 | 1 | 2 |  | 0.933954 | 0.64334 | 0.643341 |
| 0 | 1 | 0 | 0 | 1 | 1 | 2 |  | 0.79218 | 0.420551 | 0.420551 |
| 0 | 1 | 1 | 0 | 1 | 1 | 2 |  | 0.885647 | 0.627847 | 0.627848 |
| 1 | 1 | 1 | 0 | 1 | 1 | 2 |  | 0.92175 | 0.800851 | 0.800853 |
| 1 | 1 | 0 | 0 | 0 | 0 | 1 |  | 0.939529 | 0.631375 | 0.649003 |
| 0 | 0 | 1 | 0 | 0 | 0 | 1 |  | 0.861373 | 0.488805 | 0.488805 |
| 0 | 0 | 1 | 1 | 0 | 0 | 1 |  | 0.86231 | 0.448275 | 0.448275 |
| 1 | 1 | 0 | 0 | 1 | 0 | 1 |  | 0.933367 | 0.728171 | 0.728171 |
| 1 | 0 | 1 | 0 | 1 | 0 | 1 |  | 0.91811 | 0.721781 | 0.721781 |
| 1 | 1 | 1 | 0 | 1 | 0 | 1 |  | 0.940436 | 0.787325 | 0.787326 |
| 0 | 0 | 0 | 1 | 1 | 0 | 1 |  | 0.831664 | 0.378954 | 0.378953 |
| 0 | 0 | 1 | 1 | 1 | 0 | 1 |  | 0.905782 | 0.59219 | 0.592189 |
| 1 | 0 | 0 | 0 | 0 | 1 | 1 |  | 0.881597 | 0.501119 | 0.501119 |
| 0 | 1 | 0 | 0 | 0 | 1 | 1 |  | 0.817147 | 0.353872 | 0.353872 |
| 0 | 1 | 1 | 0 | 0 | 1 | 1 |  | 0.878023 | 0.506309 | 0.506309 |
| 1 | 1 | 1 | 0 | 0 | 1 | 1 |  | 0.934177 | 0.72456 | 0.724561 |
| 1 | 0 | 0 | 0 | 1 | 1 | 1 |  | 0.870852 | 0.593482 | 0.593483 |
| 0 | 0 | 1 | 0 | 1 | 1 | 1 |  | 0.858822 | 0.55025 | 0.55025 |
| 0 | 0 | 0 | 1 | 1 | 1 | 1 |  | 0.836259 | 0.428652 | 0.428651 |
| 0 | 0 | 1 | 0 | 1 | 0 | 0 |  |  |  |  |
| 0 | 1 | 1 | 0 | 1 | 0 | 0 |  |  |  |  |
| 1 | 0 | 0 | 1 | 1 | 0 | 0 |  |  |  |  |
| 1 | 1 | 0 | 1 | 1 | 0 | 0 |  |  |  |  |
| 0 | 1 | 1 | 1 | 1 | 0 | 0 |  |  |  |  |
| 1 | 1 | 0 | 0 | 0 | 1 | 0 |  |  |  |  |
| 1 | 0 | 0 | 1 | 1 | 1 | 0 |  |  |  |  |
| Note: OF, organization fit; CF, community fit; OS, organization sacrifice; CS, community sacrifice; OL, organization link; CL, community link; "~" mean absence and boolean logic "not". | | | | | | | | | | |
