# Supplementary material for: How Does the Absence of Job Embeddedness Contribute to Nurses’ Turnover Intention? A Fuzzy‐Set Qualitative Comparative Analysis
Source: J Nurs Manag. 2026 Jun 19;2026:2341935. doi: 10.1155/jonm/2341935 (PMC13282276; doi:10.1155/jonm/2341935)
Supplement: Supplementary file 3 — Supporting Information 3 Results of robustness checking configurations. [file JONM-2026-2341935-s001.docx]

**Supporting Information 3** Results of robustness checking configurations

**Table S3** Results of robustness checking configurations

| Sample set | Outcome | Configurations | Raw coverage | Unique coverage | Consistency | Solution coverage | Solution consistency |
| --- | --- | --- | --- | --- | --- | --- | --- |
| Calibration (M+SD, M, M-SD) | Turnover intention | ~OF*~OS*~CS*~OL*~CL | 0.260 | 0.022 | 0.863 | 0.485 | 0.826 |
|  |  | ~OF*CF*~OS*OL*~CL | 0.186 | 0.059 | 0.891 |  |  |
|  |  | ~OF*~OS*~CS*OL*CL | 0.209 | 0.063 | 0.833 |  |  |
|  |  | ~OF*CF*~OS*CS*~OL*CL | 0.148 | 0.023 | 0.891 |  |  |
|  |  | ~OF*~CF*~OS*~CS*~OL | 0.280 | 0.022 | 0.864 |  |  |
|  | ~Turnover intention | OF*~CF*OS*~OL*~CL | 0.196 | 0.063 | 0.886 | 0.523 | 0.858 |
|  |  | OF*~CF*OS*OL*CL | 0.199 | 0.032 | 0.901 |  |  |
|  |  | OF*CF*OS*CS*CL | 0.353 | 0.054 | 0.915 |  |  |
|  |  | CF*OS*CS*OL*CL | 0.290 | 0.015 | 0.900 |  |  |
|  |  | OF*~CF*OS*CS*OL | 0.188 | 0.009 | 0.939 |  |  |
| Frequency = 2 | Turnover intention | ~OF*CF*~OS*~CL | 0.296 | 0.046 | 0.894 | 0.536 | 0.837 |
|  |  | ~OF*~OS*CS*~OL | 0.303 | 0.027 | 0.89 |  |  |
|  |  | ~OF*~CF*~OS*~CS*CL | 0.300 | 0.082 | 0.864 |  |  |
|  |  | ~OF*~OS*~OL*~CL | 0.350 | 0.043 | 0.873 |  |  |
|  | ~Turnover intention | OF*OS*CS | 0.552 | 0.046 | 0.827 | 0.696 | 0.795 |
|  |  | OF*OS*~OL*~CL | 0.321 | 0.028 | 0.858 |  |  |
|  |  | OF*CF*CS*~OL | 0.347 | 0.021 | 0.885 |  |  |
|  |  | OF*~CF*OS*CL | 0.332 | 0.030 | 0.900 |  |  |
|  |  | OF*CF*OL*CL | 0.361 | 0.028 | 0.883 |  |  |
|  |  | OS*CS*OL*CL | 0.381 | 0.021 | 0.900 |  |  |
| Consistency > 0.9 | Turnover intention | ~OF*~CF*~OS*CS*~OL | 0.265 | 0.025 | 0.908 | 0.355 | 0.906 |
|  |  | ~OF*~CF*~OS*~OL*CL | 0.272 | 0.050 | 0.906 |  |  |
|  |  | ~OF*CF*~OS*~CS*~OL*~CL | 0.204 | 0.029 | 0.928 |  |  |
|  | ~Turnover intention | OF*OS*CS | 0.552 | 0.041 | 0.827 | 0.643 | 0.812 |
|  |  | OF*CF*CS*~OL | 0.347 | 0.010 | 0.885 |  |  |
|  |  | OF*CF*CS*CL | 0.426 | 0.015 | 0.909 |  |  |
|  |  | OS*CS*OL*CL | 0.381 | 0.021 | 0.890 |  |  |
|  |  | OF*~CF*OS*~OL*~CL | 0.267 | 0.030 | 0.891 |  |  |
| PRI > 0.7 | Turnover intention | ~OF*CF*~OS*~CS*~OL*~CL | 0.234 | 0.029 | 0.928 | 0.338 | 0.912 |
|  |  | ~OF*~CF*~OS*CS*~OL*~CL | 0.230 | 0.042 | 0.927 |  |  |
|  |  | ~OF*~CF*~OS*~CS*~OL*CL | 0.239 | 0.069 | 0.912 |  |  |
|  | ~Turnover intention | OF*OS*CS*OL | 0.407 | 0.047 | 0.870 | 0.571 | 0.833 |
|  |  | OF*CF*CS*CL | 0.426 | 0.031 | 0.909 |  |  |
|  |  | OF*OS*CS*CL | 0.451 | 0.019 | 0.901 |  |  |
|  |  | OF*~CF*OS*OL*CL | 0.276 | 0.030 | 0.905 |  |  |
|  |  | CF*OS*CS*OL*CL | 0.335 | 0.012 | 0.910 |  |  |
| Note: OF, organization fit; CF, community fit; OS, organization sacrifice; CS, community sacrifice; OL, organization link; CL, community link; "*" mean Boolean logic "and"; "~" mean absence and Boolean logic "not". | | | | | | | |
